# Expanding on *expansus*: a new species of *Scaphanocephalus* from North America and the Caribbean based on molecular and morphological data

**DOI:** 10.1017/S0031182024000647

**Published:** 2024-06

**Authors:** Sean A. Locke, Dana M. Calhoun, José M. Valencia Cruz, Erika T. Ebbs, Sandra C. Díaz Pernett, Vasyl V. Tkach, John M. Kinsella, Mark A. Freeman, Christopher A. Blanar, Pieter T. J. Johnson

**Affiliations:** 1 Departamento de Biología, Recinto Universitario de Mayagüez, Universidad de Puerto Rico, Mayagüez, Puerto Rico; 2 Department of Ecology and Evolutionary Biology, University of Colorado, Boulder, CO, USA; 3 LIMIA – IRFAP. Govern de les Illes Balears, Andratx, Balearic Islands, Spain; 4 INAGEA (UIB-CAIB), Palma, Balearic Islands, Spain; 5 Purchase College, SUNY, Purchase, NY, USA; 6 Department of Biology, University of North Dakota, Grand Forks, ND, USA; 7 Helm West Lab, Missoula, MT, USA; 8 Center for Conservation Medicine and Ecosystem Health, Ross University School of Veterinary Medicine, St. Kitts, West Indies; 9 Department of Biological Sciences, Nova Southeastern University, Fort Lauderdale, FL, USA

**Keywords:** Black Spot Syndrome, coral reef fish, fluke, helminth, raptor

## Abstract

Members of the genus *Scaphanocephalus* mature in accipitrids, particularly osprey, *Pandion haliaetus*, with metacercaria causing Black Spot Syndrome in reef fishes. In most of the world, only the type species, *Scaphanocephalus expansus* (Creplin, 1842) has been reported. Recent molecular studies in the Western Atlantic, Mediterranean and Persian Gulf reveal multiple species of *Scaphanocephalus*, but have relied on 28S rDNA, mainly from metacercariae, which limits both morphological identification and resolution of closely related species. Here we combine nuclear rDNA with mitochondrial sequences from adult worms collected in osprey across North America and the Caribbean to describe species and elucidate life cycles in *Scaphanocephalus*. A new species described herein can be distinguished from *S. expansus* based on overall body shape and size. Phylogenetic analysis of the whole mitochondrial genome of *Scaphanocephalus* indicates a close relationship with *Cryptocotyle*. We conclude that at least 3 species of *Scaphanocephalus* are present in the Americas and 2 others are in the Old World. Specimens in the Americas have similar or identical 28S to those in the Mediterranean and Persian Gulf, but amphi-Atlantic species are unlikely in light of divergence in cytochrome *c* oxidase I and the lack of amphi-Atlantic avian and fish hosts. Our results provide insight into the geographic distribution and taxonomy of a little-studied trematode recently linked to an emerging pathology in ecologically important reef fishes.

## Graphical abstract

**Figure:**
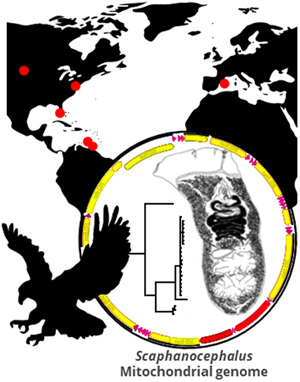

## Introduction

Members of the genus *Scaphanocephalus* (Jägerskiöld, 1904) are opisthorchiid trematodes that mature almost exclusively in osprey, *Pandion haliaetus*. Metacercariae cause Black Spot Syndrome (BSS) in fishes (Kohl *et al*., 2019; Malawauw *et al*., 2024), which manifests as immunopathogenic pigmented spots on fish skin and fins (Dennis *et al*., 2019; Kohl *et al*., 2019; Cohen-Sánchez *et al*., 2023*a*, 2023*b*). Though little studied historically, *Scaphanocephalus* is receiving increased attention, likely because BSS appears to be increasing in prevalence (Elmer *et al*., 2019; Cohen-Sánchez *et al*., 2023*a*) in keystone herbivorous fishes of importance to coral reef health (Mumby *et al*., 2007; Burkepile and Hay, 2008; Kohl *et al*., 2019), and has been linked to economic impacts in fisheries (Shimose *et al*., 2019).

Jägerskiöld (1904) named *Scaphanocephalus* for the wide forebody and narrow hindbody of adult worms that form a distinctive shape he likened to a spade. He erected the genus for a species Creplin (1842) originally described as *Monostomum expansum* from adults in osprey, having examined both Creplin's (1842) material as well as newly collected specimens from osprey in Egypt. Creplin (1842) did not mention a locality, but presumably the type specimens originated near Greifswald, Germany, where he lived and worked (Häckermann, 1876). *Scaphanocephalus expansus* has subsequently been reported from Europe, the Middle East, North America, the Gulf of Mexico, Caribbean, Japan and Malaysia (reviewed by Kohl *et al*., 2019). Two other species have been described: *Scaphanocephalus australis* (Johnston, 1916), described from the sea eagle, *Icthyophaga leucogaster*, to date endemic to Australia; and *Scaphanocephalus adamsi*, described from metacercariae found within split-level hogfish, *Bodianus mesothorax*, in the Philippines (Tubangui, 1933). Yamaguti (1942) considered *S. adamsi* a synonym of *S. expansus*, but Kifune and Kugi (1979) disagreed, based on adults of *S. adamsi* in *Buteo buteo burmanicus* from Kyushu, Japan, that presented morphological differences from *S. australis* and *S. expansus*.

Six recent studies have employed 28S rDNA to study diversity within *Scaphanocephalus* (Dennis *et al*., 2019; Kohl *et al*., 2019; Al-Salem *et al*., 2021; González-García *et al*., 2023; Cohen-Sánchez *et al*., 2023*a*, 2023*b*). Phylogenetic analysis reveals 3 28S lineages of *Scaphanocephalus* in the Gulf of Mexico, Caribbean Sea, Persian Gulf and Mediterranean (González-García *et al*., 2023). One 28S lineage occurs in both the New World and in the Persian Gulf (Al-Salem *et al*., 2021) and another is in both the New World and the Mediterranean (González-García *et al*., 2023; Cohen-Sánchez *et al*., 2023*a*, 2023*b*). The third lineage, which to date has only been found in the Gulf of Mexico and Caribbean Sea, was identified as *S. expansus* after morphological study of metacercariae (Kohl *et al*., 2019) and adults (González-García *et al*., 2023). Collectively, these works leave several questions unresolved. Firstly, available 28S sequences indicate 3 species exist where only *S. expansus* has ever been reported (González-García *et al*., 2023), indicating undescribed diversity. Second, except for 3 sequences from González-García *et al*. (2023), molecular data are from metacercariae, which impedes identification because, as in all digeneans, species of *Scaphanocephalus* are conventionally distinguished based on the adult, which differs morphologically from the metacercaria. Third, although the morphological taxonomy clearly underestimates species diversity in this genus, the additional diversity indicated by 28S may also represent an underestimate, given that ribosomal markers may be insufficiently variable to resolve recently diverged species (Vilas *et al*., 2005). Finally, the current molecular view of species distributions in *Scaphanocephalus* is perplexing in that the species recognized in recent studies as *S. expansus* (Kohl *et al*., 2019; González-García *et al*., 2023) has only been recovered in Mexico and the Caribbean, and lacks a molecular link to the Old World, from which *S. expansus* was described by Creplin (1842).

The aim of this study was to describe species and provide a robust basis for phylogenetic conclusions within *Scaphanocephalus*. Adult worms collected from across North America and the Caribbean were studied morphologically with sequencing targeted on both 28S (for comparability with past studies) and mitochondrial markers. Results were interpreted in a biogeographic context that considers the distribution of hosts and geographic barriers. Whole mitochondrial genome and nuclear ribosomal operon data were also obtained to clarify the phylogenetic placement of the genus *Scaphanocephalus* and to provide resources for future molecular phylogenetic work.

## Materials and methods

### Sample collection and processing

Specimens of *Scaphanocephalus* were newly collected from salvaged osprey carcasses (provided by wildlife rehabilitation centres) and from ocean surgeonfish (*Acanthurus tractus*) collected in North America and the Caribbean (Table 1, Supplementary Table 1). Adult worms were removed from osprey gastrointestinal tracts while metacercariae were mechanically excysted from fins and stored in 95% ethanol until molecular or morphological analysis. In specimens or subsamples of worms intended for molecular work, DNA was extracted using commercial kits following manufacturer instructions (e.g. NucleoSpin Tissue XS, Macherey Nagel, Allentown, PA, USA). Vouchers for morphological study were gradually rehydrated, stained with acetocarmine, gradually dehydrated in pure alcohol, cleared in clove oil and mounted on slides using Permount. Voucher specimens were deposited in the Museum of Southwestern Biology (MSB:Para:49134–46). Line drawings of adults were made with a Nikon Alphaphot YS equipped with a camera lucida.

**Table 1.** Specimens of *Scaphanocephalus* sequenced in the present and prior studies

Species	GenBank accessions								
Developmental stage (n)	CO1 BC	CO1 JB3	28S	MT genome	rDNA operon	Museum vouchers	Host	Locality	Source
*Scaphanocephalus robustus* n. sp.									
Adult (5)		PP456658–9, PP456666–8	PP436423–4, PP436427–9			MSB:Para:49134–5, 49139–40	*Pandion haliaetus*	Curaçao	Present study
Adult (11)		PP456660–5, PP456676–9	PP436426, PP436430–4			MSB:Para:49141–2, 49146	*P. haliaetus*	Florida	Present study
Adult (7)	PP456673, PP456675	PP456669–75	PP436425, PP436435–7			MSB:Para:49136–8, 49143–5	*P. haliaetus*	Virginia	Present study
Metacercaria (1)			OR794154				*Mugil curema*	Celestún, Yucatán, Mexico	González-García *et al*. (2023)
*Scaphanocephalus* sp. ne2									
Adult (1)	PP456684	PP456683					*P. haliaetus*	Florida	Present study
Adult (1)	PP456682		PP436440				*P. haliaetus*	Montana	Present study
Metacercariae (10)			MN160569				*Acanthurus bahianus, A. coeruleus, A. chirurgus*	St. Kitts	Dennis *et al*. (2019)
*Scaphanocephalus* sp. ne1									
Adult (1)				PP577105	PP430581		*P. haliaetus*	Florida	Present study
Metacercariae (2)	PP456680	PP456680–1	MN160570				*A. chirurgus*	St. Kitts	Present study, Dennis *et al*. (2019)
Metacercariae (2)			PP436438–9				*A. bahianus*	Curaçao	Present study
Metacercariae^a^ (2)			MK680936				*Sparisoma chrysopterum*	Bonaire	Kohl *et al*. (2019)
Adult (3)^a^			OR794151–3				*P. haliaetus*	Campeche, Mexico	González-García *et al*. (2023)
*Scaphanocephalus* sp. pa1									
Metacercariae (8)	OR075091		OK045681–8				*Xyrichtys novacula*	Balearic Islands, Mediterranean	Present study, Cohen-Sánchez *et al*. (2023*a*, 2023*b*)
*Scaphanocephalus* sp. pa2									
Metacercariae (5)			MT461356				*Siganus argenteus*	Persian Gulf, Saudi Arabia	Al-Salem *et al*. (2021)

CO1 BC = barcode region of cytochrome *c* oxidase I, CO1 JB3 = 3′ region of cytochrome *c* oxidase I, 28S = partial sequence of 28S nuclear rDNA; MT genome = whole mitochondrial genome; rDNA operon = nuclear rDNA array, with partial external transcribed spacer; MSB = Museum of Southwestern Biology. A more detailed version of the information in this table is in Supplementary Table 1.

a Identified as *Scaphanocephalus expansus* by study authors.

Extracted DNA from prior studies (Dennis *et al*., 2019; Cohen-Sánchez *et al*., 2023*b*) was also re-analysed in the present study. These extracts were from (1) a metacercaria (specimen XN4P4M) from a pearly razorfish (*Xyrichtys novacula*) caught offshore from the Balearic Islands in the Mediterranean Sea, from which Cohen-Sánchez *et al*. (2023*b*) obtained 28S sequence OK045682, and (2) 2 specimens from a doctorfish (*Acanthurus chirurgus*) in St. Kitts, from which Dennis *et al*. (2019) obtained 28S sequence MN160570.

### Molecular analysis

#### Next generation sequencing

The DNA of an adult worm from a salvaged osprey from Florida was extracted and shotgun-sequenced on an Illumina HiSeq 4000 at Azenta (NJ, USA). Nuclear ribosomal DNA operon and whole mitochondrial genomes were assembled using Geneious Prime v2020.2.2 (Biomatters Ltd., Auckland NZ) from 150-bp paired-end reads built with Nextera adapters (Illumina, San Diego, CA, USA). The nuclear rDNA operons were iteratively assembled using custom settings with medium-low sensitivity read-mapping to a chimeric consensus of 2 28S sequences of *Scaphanocephalus* (MN160569, MN160570) and the entire rDNA operon of *Diplostomum ardeae* (MT259036) until even and deep coverage of the assembly was obtained. The boundaries of rDNA subunit genes and the transcribed spacers were determined by aligning the assembly with other sequences (MH521249–52, Locke *et al*., 2018). Mitochondrial genomes were assembled by mapping Illumina reads to a consensus of 2 mitochondrial genomes from Heterophyidae: *Haplorchis taichui* (MG972809) and *Metagonimus yokogawai* (KC330755). The longest resulting fragment with good coverage was then extended in iterative assemblies until the molecule was circularized. Coding regions, transfer RNAs (tRNAs) and ribosomal RNAs (rRNAs) were annotated using MITOS (Bernt *et al*., 2013) and through comparison with KC330755 (unpublished), EU921260 and FJ381664 (Shekhovtsov *et al*., 2010), KC330755 (unpublished), KT239342 (Na *et al*., 2016), MG972809 (Le *et al*., 2021), MH536507–13 (Locke *et al*., 2018) and NC_063968 (unpublished).

As described in the results, 2 specimens (1 from Montana, 1 from Florida) each yielded CO1 Sanger sequences that differed by a large and similar magnitude from all other sequences obtained. The specimens were suspected to belong to the same species, but their CO1 sequences could not be compared because different regions of this gene were amplified in each. After unsuccessful attempts to amplify and Sanger-sequence CO1 fragments allowing direct comparison, extracted DNA from the Florida specimen was subject to Minion sequencing with the goal of obtaining CO1 sequence comparable to the Montana specimen. The sequencing library was prepared using the Ligation Sequencing Kit v14 (SQK-LSK114, Oxford Nanopore Technologies, Oxford, UK) following the manufacturer's protocol. The library was sequenced on a whole Flongel Flow Cell (R10.4.1, Oxford Nanopore Technologies) with MinKnow v.21.05.12 software (Oxford Nanopore Technologies). Base-calling was performed in Guppy (v.5.0.12). NanoFilt v.2.8.0 (De Coster *et al*., 2018) was used to process and filter all resulting reads with quality scores ≥Q10. MiniMap2 v.2.22 (Li, 2018) was used to remove non-trematode contaminant reads (host, human, bacteria).

#### Sanger sequencing

Partial 28S was amplified using the primers LSU5 (Littlewood, 1994) and 1500R (Snyder and Tkach, 2001) with the following thermocycling conditions: 95°C for 30 s, followed by 30 cycles of 95°C for 30 s, 56°C for 45 s and 68°C for 60 s; and a final incubation at 68°C for 5 min. The 5′ or barcode region of the mitochondrial cytochrome *c* oxidase I (CO1) gene was amplified using the primers and protocols MplatF/R, MplatF/Dice11 and MplatF/Dice14 of Moszczynska *et al*. (2009) and Van Steenkiste *et al*. (2015), using the touchdown protocol described by Van Steenkiste *et al*. (2015). The 3′ half of CO1 was amplified using primers JB3 (Morgan and Blair, 1998) and CO1-R (Miura *et al*., 2005) using the cycling conditions of Miura *et al*. (2005).

#### Phylogenetic analysis

Sequences generated in the present study were aligned with data from the literature and discovered through BLAST searches (Altschul *et al*., 1997) using MAFFT (Katoh and Standley, 2013; Supplementary Table 1). Uncorrected *P*-distances were calculated using all alignment sites in MEGA X v.10.1.8 (Kumar *et al*., 2018). Phylogenetic trees based on alignments stripped of gaps were based on substitution models selected using the Bayesian Information Criterion in MEGA X v.10.1.8 (Kumar *et al*., 2018), or the nearest approximation available in maximum likelihood (ML) (RAxML, Stamatakis, 2014) and Bayesian inference (BI, Huelsenbeck and Ronquist, 2001) implemented in Geneious. Phylogenetic accuracy in ML was estimated by bootstrapping the trees with 1000 replicates and in BI, with 2 runs of total chain length 1 100 000, subsampled every 200 generations, a burn-in length of 110 000, yielding 4951 trees.

## Results

Molecular data were obtained from 27 newly collected adult *Scaphanocephalus* specimens found within osprey from Montana, Virginia, Florida and Curaçao, and 2 metacercariae from newly collected ocean surgeonfish (*A. tractus*) from Curaçao (Table 1). Mitochondrial data were also newly obtained from 3 specimens from which 28S was sequenced in prior studies, namely 2 metacercariae from St Kitts (specimens QSK29.1, QSK29.2, 28S sequence MN160570 from Dennis *et al*. [2019]) and 1 from the Spanish Mediterranean (specimen XN4P4M, 28S sequence K045682 from Cohen-Sánchez *et al*. [2023*b*]). As presented further below and summarized in Table 2, molecular and morphological data supported description of a new species. As many as 4 other putative species were not described, identified or characterized morphologically due to a lack of vouchers of adult worms or of CO1 sequences: *Scaphanocephalus* sp. ne1, *Scaphanocephalus* sp. ne2, *Scaphanocephalus* sp. pa1, *Scaphanocephalus* sp. pa2 (where ‘ne’ refers to Nearctic and ‘pa’ to Palearctic). Sequence-based identifications revealed mixed infections in osprey. One osprey from Florida was infected with 6 *S. robustus* n. sp. and 1 *Scaphanocephalus* sp. ne2, and a second osprey from Florida was infected with 4 *S. robustus* n. sp. and 1 *Scaphanocephalus* sp. ne1.

**Table 2.** Summary of species distributions in the present and past studies based on morphological and molecular (cytochrome *c* oxidase I, CO1; partial nuclear 28S rDNA, 28S) data

	Hosts	Geographic distribution	Morphological and distributional support for species	Molecular support for species	Source
*Scaphanocephalus robustus* n. sp.	*Pandion haliaetus, Mugil curema*	Curaçao; Florida, Virginia, USA; Yucatán, Mexico	Smaller and with greater hindbody width/total length ratio greater than in *S. expansus* from type region. Eggs slightly longer, narrower, and testes more deeply lobed than *S. australis*. Unlikely to be conspecific with Old World species	Divergence in CO1 from all other species; divergence in 28S from all other species except *Scaphanocephalus* sp. pa1; phylogenetically separate lineage from *Scaphanocephalus* sp. pa1 (Fig. 5)	Present study; González-García *et al*. (2023)
*Scaphanocephalus* sp. ne2	*P. haliaetus*, *Acanthurus tractus*, *A. coerulus*, *A. chirurgus*	St. Kitts; Montana, USA	Unlikely to be conspecific with Old World species	Divergence in 28S from all other species except *Scaphanocephalus* sp. pa2. Divergence in CO1 from all other species (CO1 not available from *Scaphanocephalus* sp. pa2). Phylogenetically separate lineage according to CO1, but not 28S (identical in *Scaphanocephalus* sp. pa2)	Present study, Dennis *et al*. (2019)
*Scaphanocephalus* sp. ne1	*P. haliaetus*, *Acanthurus tractus*, *A. coerulus*, *Sparisoma chrysopterum*	Florida, USA; St. Kitts; Curaçao; Campeche, Mexico	Egg length smaller than in *S. robustus* n. sp. Body total length, anterior width and posterior width generally smaller than in Old World descriptions of *S. expansus*. Unlikely to be conspecific with Old World species	Divergence and phylogenetic lineage independence in CO1 and 28S from all other species	Present study, Dennis *et al*. (2019); Kohl *et al*. (2019); González-García *et al*. (2023)
*Scaphanocephalus* sp. pa1	*Xyrichtys novacula*	Mediterranean	Unlikely to be conspecific with New World species	Divergence in CO1 from all other species; divergence in 28S from all other species except *S. robustus* n. sp.; phylogenetically separate lineage from *S. robustus* n. sp. according to concatenated 28S + CO1, but not if 28S/CO1 analysed separately	Present study; Cohen-Sánchez *et al*. (2023*b*)
*Scaphanocephalus* sp. pa2	*Siganeus argentus*	Persian Gulf	Unlikely to be conspecific with New World species	Divergence in 28S, and phylogenetically independent lineage from all other species except *Scaphanocephalus* sp. ne2, which shares identical 28S. No CO1 available from *Scaphanocephalus* sp. pa2	Al-Salem *et al*. (2021)

**Figure 1.**
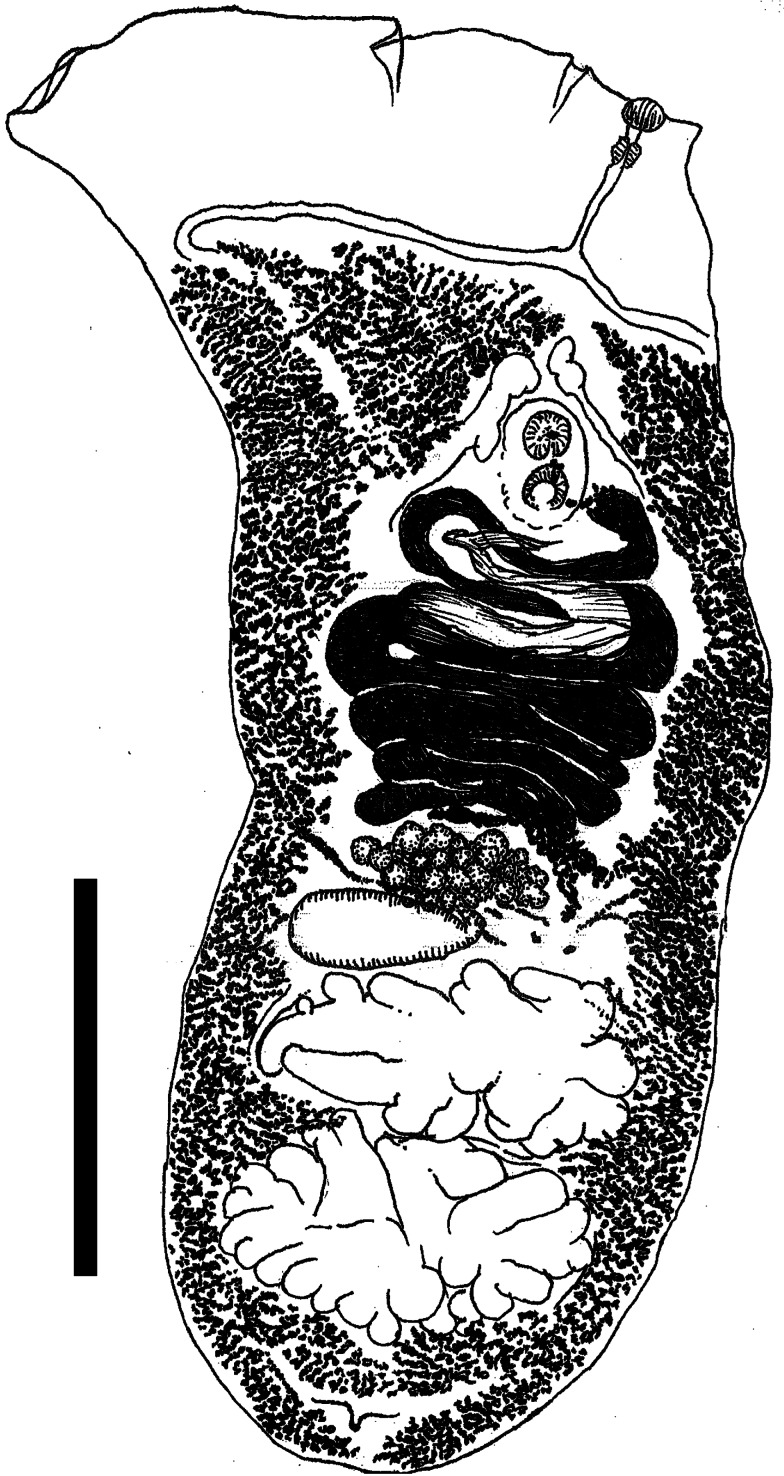
Line drawing of type specimen of *Scaphanocephalus robustus* n. sp. from *Pandion halietus* from Virginia, USA. Hologenophore for sequence PP456670 (cytochrome *c* oxidase I) and PP436435 (28S), deposited in the Museum of Southwestern Biology (MSB:Para:49138). Scale bar is 1 mm.

**Figure 2.**
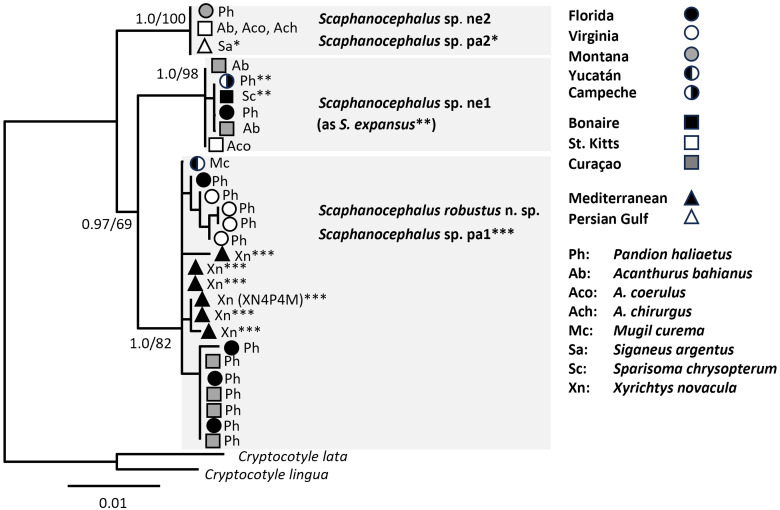
Phylogenetic analysis of 28 partial 28S sequences of *Scaphanocephalus* in the present (16 sequences) and prior studies (12 sequences). The maximum likelihood topology is shown and nodes are annotated with posterior probability (Bayesian inference)/bootstrap support (1000 replicates in maximum likelihood). The trimmed alignment was 1048 nt in length. The ML tree was generated using nucleotide substitution model GTR + G; the BI tree using HKY + G. The same tree, with individual sequences labelled with GenBank accessions, is available in Supplementary Fig. 1.

**Figure 3.**
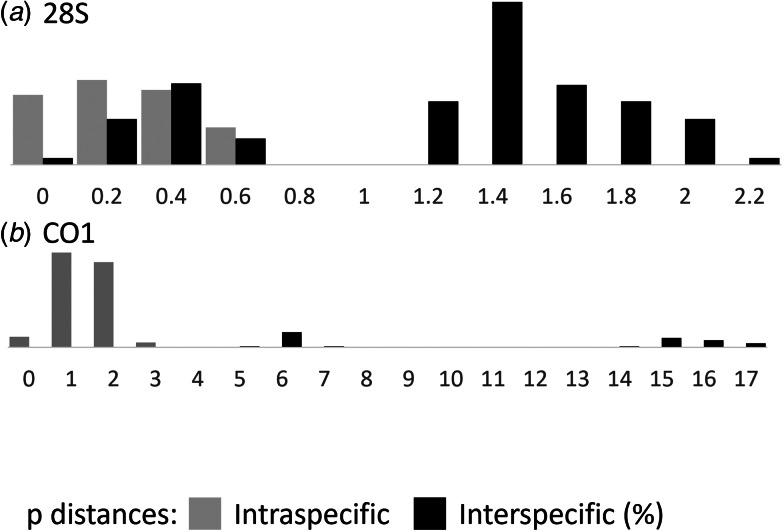
Histogram of uncorrected *P* distances in *Scaphanocephalus* among (a) 33 partial 28S sequences from the present and prior studies and (b) 28 partial cytochrome *c* oxidase 1 sequences from the present study.

**Figure 4.**
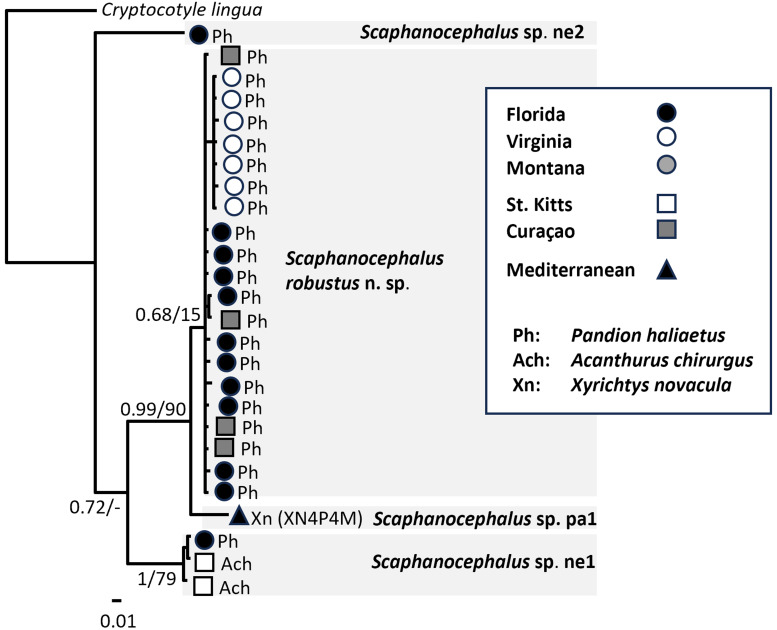
Phylogenetic analysis of 26 partial CO1 sequences of *Scaphanocephalus* generated in the present study. The Bayesian inference topology is shown with nodes annotated with posterior probability (Bayesian inference, BI)/bootstrap support (1000 replicates in ML) based on a 415-nt alignment. The ML tree was generated using substitution model GTR + I; the BI tree using HKY + I.

**Figure 5.**
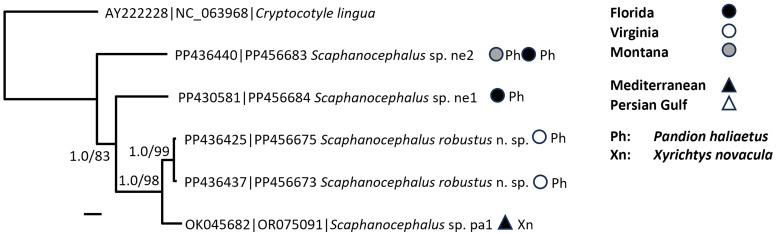
Phylogenetic analysis of concatenated partial 28S (1059 nt) and CO1 (374 nt) sequences of *Scaphanocephalus*. The maximum likelihood topology is shown and nodes are annotated with posterior probability (Bayesian inference)/bootstrap support (1000 replicates in maximum likelihood). The ML tree was generated using substitution model GTR + I; the BI tree using HKY + I with unlinked parameters in an alignment partitioned into 28S and CO1.

**Figure 6.**
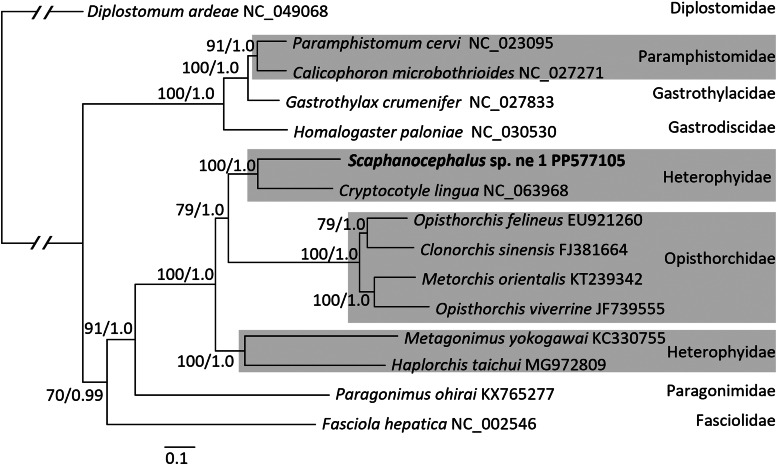
Phylogenetic analysis of complete mitochondrial genomes (1059 nt). The maximum likelihood topology is shown and nodes are annotated with posterior probability (Bayesian inference)/bootstrap support (1000 replicates in maximum likelihood). Maximum likelihood and Bayesian inference trees were both generated using substitution model GTR + G + I.

Detailed presentation of morphological and molecular support for species is in the Results and Tables 3 and 4 and Figs 1–6.

### Description

#### *Scaphanocephalus robustus* n. sp.

Type host: *Pandion haliaetus*; other hosts: *Mugil curema*

Type locality: Virginia, USA; other localities: Florida, USA; Curaçao; Celestún, Yucatán, Mexico

Voucher specimens: MSB:Para:49134–46

GenBank: 28S: PP436427–34, OR794154; CO1: PP456658–80

Description based on 15 hologenophores from *P. haliaetus* (Table 3, Fig. 1): Dorsoventrally flattened, with wide lateral expansions in forebody; hindbody subcylindrical. Oral sucker small, subterminal. Short pre-pharynx followed by small pharynx and lengthy oesophagus. Muscular genital complex (ventral sucker, gonotyl) in anterior third of body. Vitellaria in 2 lateral extracaecal fields, confluent posteriorly in most specimens, extending anteriorly to caecal bifurcation. Ovary multilobate, median, pretesticular; testes tandem, deeply lobed, in posterior third of body. Seminal receptacle submedian, elongate, extending laterally between ovary and anterior testis.

**Table 3.** Morphometrics of *Scaphanocephalus* described in the present and prior studies, as mean (range), ±standard deviation, *n* measured, in *μ*m

	*Scaphanocephalus robustus* n. sp.	*Scaphanocephalus* sp. ne1 (as *S. expansus*)	*S. expansus*	*S. expansus*	*S. expansus*	*S. expansus*	*S. australis*	*S. adamsi*	*S. adamsi*
	Current study	González-García *et al*. (2023)	Creplin (1842)	Jägerskiöld (1904)	Dubois (1960)	Foronda *et al*. (2009)	Johnston (1916)	Tubangui (1933)	Kifune and Kugi (1979)
Location	Virginia, Florida, USA; Curaçao	Campeche, Mexico	Germany	El Tor, Egypt	Sète, France	Canary Islands, Spain	Terrigal, Australia	Manilla, Philippines	Oita, Kyushu, Japan
Host species	*P. haliaetus*	*P. haliaetus*	*P. haliaetus*	*P. haliaetus*	*P. haliaetus*	*P. haliaetus*	*Icthyophaga leucogaster*	*Bodianus mesothorax*	*Buteo buteo burmanicus*
Life stage	Adult	Adult	Adult	Adult	Adult	Adult	Adult	Metacercaria	Adult
*N*	15	19	4		3	43?	3		≈10
Length	3340 (2178–4178) ± 646, 9	(2256–5063)	5569 (4794–6204)	5000	(4650–4760)	3261 ± 449	(3000–3250)	(3020–3070)	(3500–3840)
Anterior width	2725 (2376–3248) ± 302, 7	(1177–2792)	3948	3200 (1000–1500 unextended)	(2730–3290)	1690 ± 455	2200	(2150–2370)	(2920–3560)
Posterior length	2391 (1386–3406) ± 599, 11		4230						
Posterior width	1047 (634–1366) ± 238, 14	(643–1375)	1410	1000	–	926 ± 126	1170	(1170–1210)	(1510–1760)
Oral sucker length	98 (77–117) ± 13, 6	(64–129)		128	138	101 ± 6	134	120	(110–150)
Oral sucker width	108 (71–133) ± 27, 4	(74–116)		96	125	86 ± 3	107	(110–130)	(100–140)
Prepharynx length	22 (8–40) ± 16, 4	(14–43)			(37–42)	84 ± 12			
Pharynx length	76 (55–85) ± 11, 6	(54–88)		80	(81–95)	76 ± 7	96	(80–90)	(95–110)
Pharynx width	61 (38–69) ± 12, 6	(40–87)		60	(78–85)	58 ± 12	75	80	(70–100)
Oesophagus	167 (83–250) ± 66, 6	(151–309)		272	180	215 ± 52	270	(60–110)	(80–140)
Ventrogenital length	288 (198–359) ± 55, 10	(214–415)		270			276	(180–210)	
Ventrogenital width	198 (109–273) ± 52, 11	(178–279)					214	(130–190)	
Ovary length	159 (55–396) ± 98, 13	(105–263)		160	(270–380)	171 ± 37	155	(100–110)	(160–180)
Ovary width	388 (203–535) ± 109, 13	(226–454)		400	(700–870)	298 ± 75	407	(270–280)	(600–660)
Seminal receptacle length	120 (47–178) ± 50, 15					105 ± 37			
Seminal receptacle width	381 (139–625) ± 154, 15					1498 ± 227			
Anterior testis length	399 (234–772) ± 141, 15	(221–465)		(480–560)	(440–690)	375 ± 74		(190–230)	(420–630)
Anterior testis width	734 (422–970) ± 183, 15	(442–912)		(740–860)	(1270–1300)	593 ± 72		(740–760)	(1060–1220)
Posterior testis length	421 (198–609) ± 123, 15	(276–495)		(480–560)	(580–840)	428 ± 89		(200–270)	(560–780)
Posterior testis width	739 (453–1069) ± 182, 15	(424–951)		(740–860)	(1150–1270)	634 ± 86		(720–800)	(1200–1340)
Egg length	32 (28–34) ± 2.2, 13	(22–24)		27	29 (22–40)	25 ± 5.5	29 (24–32)		(34–36)
Egg width	18 (14–20) ± 1.9, 13	(18–21)		16	17 (16–20)	18 ± 3.8	20 (19–21)		(17–18)
Excretory pore to posterior margin	65 (38–102) ± 22, 6						(70–130)

#### Remarks

Compared with descriptions of *S. expansus* originating in the Old World by Creplin (1842), Jägerskiöld (1904) and Dubois (1960), *S. robustus* n. sp. is shorter in total length, with a relatively wide hindbody. In *S. robustus* n. sp., the width of the hindbody is mean 34% (range 26–46 ± 7%) of total length, while in descriptions of *S*. *expansus* from the Old World, this proportion is mean 25%, range 23–29% (Creplin, 1842), 20% (Jägerskiöld, 1904) and 28% (Foronda *et al*., 2009). The relative width of the hindbody in adults of *S. adamsi* described by Kifune and Kugi (1979), from 44 to 46% of total length, was greater than in *S. robustus* n. sp. Foronda *et al*. (2009) described worms identified as *S*. *expansus* that were similar in length and proportion to *S. robustus* n. sp. but with smaller eggs.

Among New World species, *S. robustus* n. sp. can be distinguished from *Scaphanocephalus* sp. ne1, described by González-García *et al*. (2023) as *S. expansus*, by the longer eggs and, to a lesser extent, the wider anterior body of *S. robustus* n. sp. Morphometrically, *S. robustus* n. sp. most closely resembles *S. australis*, which Johnston (1916) described from *I. leucogaster* in Australia, but in *S. robustus* n. sp. eggs are slightly longer and narrower, and testes are deeply lobed, rather than solid bodies.

Although *S. robustus* n. sp. is morphologically distinguished from *S. expansus* based largely on metrical variation, caution is needed in making metrical comparisons among descriptions because authors have reported the range, both mean and range, mean and standard deviation or unexplained figures, often without specifying the number of specimens in which a feature was measured. Notably, most measurements provided by Jägerskiöld (1904), who examined the type specimens of Creplin (1842), are inconsistent with or lack the variability of the original description. Foronda *et al*. (2009) reported the seminal receptacle to be wider than the hindbody, with a large standard deviation suggesting this was not a typographical error. González-García *et al*. (2023) apparently summed the standard deviations to the means to obtain a range of measurements in Foronda *et al*. (2009), thus underestimating morphometric variation in the latter study.

### Phylogenetic results: Sanger sequencing

#### 28S rDNA

The 19 new sequences of 28S generated in this study were aligned with 16 sequences collectively published by Kohl *et al*. (2019), Dennis *et al*. (2019), Al-Salem *et al*. (2021), Cohen-Sánchez *et al*. (2023*b*) and González-García *et al*. (2023). Phylogenetic analysis yielded 3 strongly supported clades (Fig. 2). The largest clade consisted of *S. robustus* n. sp. (from North America, the Caribbean and the Yucatán), with sequences from the Mediterranean nested within (including OK045682 from specimen XN4P4M, from which divergent CO1 was newly obtained in the present study, see below). Another 28S clade was formed by identical sequences from specimens from the Persian Gulf, and Montana and the Caribbean. A third clade, *Scaphanocephalus* sp. ne1, was formed by 28S from specimens collected in the Gulf of Mexico and Caribbean (Florida, St. Kitts, Curaçao, Bonaire, Campeche), including specimens identified as *S. expansus* by Kohl *et al*. (2019) and González-García *et al*. (2023).

Genetic distances among 28S sequences of the genus *Scaphanocephalus* were bimodally distributed, with a lack of *P*-distances greater than 0.57% and less than 1.01% (Fig. 3). This gap in 28S *P* distances corresponded to intra and inter-clade variation in 28S (Fig. 2), but not to comparisons within and between species, as recognized herein. For example, 28S distances between *S. robustus* n. sp. and *Scaphanocephalus* sp. pa1 averaged 0.27 (range 0–0.57%) and thus were similar in magnitude to intraspecific 28S distances in *Scaphanocephalus* (Table 4).

**Table 4.** Average genetic distances (range in parenthesis) within and among species of *Scaphanocephalus* in cytochrome *c* oxidase I (CO1) and 28S rDNA (uncorrected *P* expressed as per cent difference among all sites in alignments of 29 sequences of CO1, 33 of 28S)

	*Scaphanocephalus* sp. ne1	*Scaphanocephalus* sp. pa1	*Scaphanocephalus* sp. pa2	*Scaphanocephalus robustus* n. sp.	*Scaphanocephalus* sp. ne2	Overall
CO1 intraspecific				0.92 (0–2.54)	0 (0–0)	0.92 (0–2.54)
CO1 interspecific	15.04 (13.49–16.62)	6.6 (5.0–16.74)		12.68 (5.0–17.57)	17.2 (16.32–17.76)	12.84 (5.0–17.76)
28S intraspecific	0.0489 (0–0.2318)	0.1507 (0–0.3897)		0.2205 (0–0.5208)	0 (0–0)	0.1835 (0–0.5208)
28S interspecific	1.3541 (1.1008–2.0481)	0.7203 (0–2.0796)	1.5805 (0–1.9531)	1.0169 (0–2.0735)	1.6998 (0–2.0796)	1.1264 (0–2.0796)

#### Cytochrome *c* oxidase I

Phylogenetic analysis of partial CO1 revealed 4 lineages with variable node support (Fig. 4). The topology of the CO1 phylogeny was consistent with the 28S phylogeny (Fig. 2) in that *Scaphanocephalus* sp. ne2 was the earliest diverging lineage, its ancestor giving rise to other clades, including *Scaphanocephalus* sp. ne1, which emerged as reciprocally monophyletic in analyses of both markers. The CO1 phylogeny (Fig. 4) differed from the 28S phylogeny (Fig. 2), in that CO1 from the Mediterranean specimen of *Scaphanocephalus* sp. pa1 (XN4P4M) was separate from New World specimens of *S. robustus* n. sp., albeit with weak support. A concatenated alignment of 28S and CO1 generated to clarify the phylogenetic position and status of this Mediterranean specimen (XN4P4M) with respect to *S. robustus* n. sp. showed *Scaphanocephalus* sp. pa1 to be separate from the *S. robustus* n. sp. in both BI and ML (Fig. 5).

Among the 4 species (*Scaphanocephalus* spp. ne1, ne2, pa1 and *S. robustus* n. sp.) from which CO1 sequences were available, *P*-distances were as high as 2.54% within and at least 5.0% between species (Fig. 3, Table 4). Between the most closely related species (*S. robustus* n. sp., *Scaphanocephalus* sp. pa1), *P* distances averaged 5.5% (range 5.0–6.2%) and the 59th translated amino acid (glycine) from the single specimen of *Scaphanocephalus* sp. pa1 differed from the amino acid found in all specimens in the New World (alanine). Under the alternative interpretation that *Scaphanocephalus* sp. pa1 and *S. robustus* n. sp. represent a single, transatlantic species, CO1 variation within this species would average 1.27 (range 0–6.2%).

### Phylogenetic results: next generation sequencing

#### Nuclear rDNA operon

Of 73 675 730 150-bp Illumina reads obtained from the DNA of an adult *Scaphanocephalus* from *P. haliaetus* in Fort Lauderdale, Florida, 52 581 were assembled to an rDNA operon contig 7571 bp long, with mean coverage of 1015 reads per site (range 477–1930) over subunit and internal transcribed spacer sites (GenBank accession: PP430581). The guanine + cytosine (G + C) content of the rDNA operon was slightly enriched (52.4%) and the 18S subunit was 1991 nt long, ITS1 798 nt, 5.8S 157 nt, ITS2 275 and 28S 4194 nt. Blast searches using the entire rDNA operon or portions thereof yielded top hits belonging to *Cryptocotyle lingua*, with overall operon similarity of 7194/7618 identities, or 94.4% similarity with *C. lingua* (MW361240), including 2 large gaps (20 and 162 nt) in the ITS1 portion of the alignment.

#### Mitochondrial genome

The mitochondrial genome assembly of *Scaphanocephalus* sp. ne1 (GenBank accession: PP577105) was a circular molecule 14 188 bp in length, with mean of 2279 (range 516–17 204) reads per site from the 5′ end of cox3 to the 3′ end of nad5 (i.e. excluding a difficult to assemble, non-coding, region repetitive that artefactually increases read depth). Annotations yielded 36 genes: 12 protein-coding genes (*cox1*-*3*, *nad1*-6, *nad4L*, *atp6*, *cob*), 22 tRNA genes and 2 rRNA genes (Table 5) transcribed in the same direction (5′–3′). Mitochondrial coding genes were separated by short intergenic sequences except for *cox2* and *nad6*, which overlap by 17 bp. The start codon ATG was used in 7 protein-coding genes, followed by GTG codon in 4 genes, and TTG in 1, and all coding genes ended in the TAG stop codon except *nad2*, which terminated with TAA. The molecule was A + T enriched, like other opisthorchiid mt genomes (Table 6).

**Table 5.** Position and characteristics of protein-coding and non-coding sequences from the mt genome of *Scaphanocephalus* sp. ne 1 (GenBank accession: PP577105)

		Coding position			Codon
Name	Type	Start	End	Length	Start/stop
*cox3*	CDS	1	648	648	ATG/TAG
*trnH*	tRNA	657	727	71	
*Cytb*	CDS	739	1848	1110	ATG/TAG
*nad4l*	CDS	1853	2119	267	GTG/TAG
*nad4*	CDS	2080	3366	1287	ATG/TAG
*trnQ*	tRNA	3375	3437	63	
*trnF*	tRNA	3490	3555	66	
*trnM*	tRNA	3555	3625	71	
*atp6*	CDS	3626	4141	516	ATG/TAG
*nad2*	CDS	4213	5082	870	ATG/TAA
*trnV*	tRNA	5094	5167	74	
*trnA*	tRNA	5208	5269	62	
*trnD*	tRNA	5300	5364	65	
*nad1*	CDS	5365	6267	903	GTG/TAG
*trnN*	tRNA	6274	6344	71	
*trnP*	tRNA	6346	6413	68	
*trnI*	tRNA	6418	6482	65	
*trnK*	tRNA	6493	6559	67	
*nad3*	CDS	6562	6918	357	GTG/TAG
*trnS*	tRNA	6970	7030	61	
*trnW*	tRNA	7043	7111	69	
*cox1*	CDS	7118	8656	1539	TTG/TAG
*trnT*	tRNA	8657	8722	66	
*16S rrnL*	rRNA	8723	9706	984	
*trnC*	tRNA	9707	9770	64	
*12S rrnS*	rRNA	9778	10 527	750	
*cox2*	CDS	10 528	11 199	672	ATG/TAG
*nad6*	CDS	11 183	11 644	462	ATG/TAG
*trnY*	tRNA	11 647	11 711	65	
*trnL1*	tRNA	11 713	11 779	67	
*trnS2*	tRNA	11 777	11 846	70	
*trnL2*	tRNA	11 861	11 926	66	
*trnR*	tRNA	11 961	12 030	70	
*nad5*	CDS	12 031	13 614	1584	GTG/TAG
*trnE*	tRNA	13 654	13 721	68	
*trnG*	tRNA	14 027	14 095	69

**Table 6.** Comparison of mitochondrial genomes from the Opisthorchiidae

Species	GenBank accession number	Base composition (%)					Total bp length	Total number of codons
		A	C	G	T	A + T		
*Scaphanocephalus* sp. ne 1	PP577105	16.0	12.7	26.8	44.5	60.5	10 215	3405
*Cryptocotyle lingua*	NC_063968	20.5	11.5	22.2	45.8	66.3	10 173	3391
*Haplorchis taichui*	MG972809	17.0	11.9	27.9	43.1	60.2	10 164	3360
*Metagonimus yokogawai*	KC330755	15.3	14.3	29.7	40.7	56	10 245	3415
*Opisthorchis felineus*	EU921260	15.3	12.1	27.2	45.3	60.7	10 218	3106
*Metorchis orientalis*	KT239342	14.4	11.4	26.9	47.2	61.6	10 176	3392
*Clonorchis sinensis*	FJ381664	15.7	11.9	27.3	45.1	60.8	10 209	3403

In phylogenetic analysis of mitochondrial genomes, *Scaphanocephalus* and *Cryptocotyle* emerged as sisters while the families Opisthorchidae and Heterophyidae were not monophyletic (Fig. 6).

Minion sequencing was used to resolve the status of 2 adult specimens from 2 salvaged osprey that yielded Sanger sequences from different parts of the CO1 gene (Supplementary Fig. 3). The *P* distances of the barcode CO1 Sanger sequence (3′ region, PP456682) from a specimen of *Scaphanocephalus* sp. ne2 from Montana averaged 16.5% (range 16.3–16.6%) to CO1 from all other specimens, while the 3′ CO1 Sanger sequence (PP456683) of a worm from Florida averaged 17.2% (range 16.3–17.8%). However, these 2 Sanger sequences did not overlap and could not be compared, and repeated attempts to amplify and sequence CO1 fragments allowing direct comparison were unsuccessful. Minion sequencing of the Florida specimen yielded 27 411 reads (average length of 2618 nt) and 7457 contigs averaging 2751.1 (range 115–6963, s.d. ± 1200.7) nt in length, some assembled from hundreds of reads. Most (95%) were filtered out as contamination after mapping to bacterial, human or avian scaffolds. The remaining 356 contigs were mean 593.3, range 165–2370, s.d. ± 306.0 in length. Of the latter, a 198-nt contig mapped to the CO1 sequence obtained from Montana with 90.2% similarity. All variation between the Minion contig of the Florida specimen and the Montana specimen occurred in the first (5′) 47 and last (3′) 29 nt; the central 122 nt (PP456684) were identical in the Minion contig and the PCR-amplified, Sanger-sequenced CO1 of *Scaphanocephalus* sp. ne2 (PP456682) from Montana. Quality scores of the Minion contig were lower in these 5′ and 3′ marginal regions (mean quality 15.0, range 3–43, s.d. ± 8.8), and trimmed by default parameters in Geneious. In contrast, the central 122 bp of the Minion contig identical to the Sanger sequence of *Scaphanocephalus* sp. ne2 had mean quality 26.7, range 6–90, s.d. ± 13.1. We interpret these results as indicating the Florida specimen also belongs to *Scaphanocephalus* sp. ne2.

## Discussion

Here we report a large-scale, molecular phylogenetic study of the trematode genus *Scaphanocephalus*. We describe a new species and find evidence for 4 others (2 in North America and 2 in Europe), ultimately concluding that none of these 4 can be reliably identified as *S. expansus* based on currently available data. We provide mitogenomic confirmation of the close relationship between this genus and *Cryptocotyle*, previously suspected based on morphology and phylogenetic analysis of a smaller number of nuclear characters. The genomic resources provided here will be useful in ongoing and future work that is needed in view of the association of *Scaphanocephalus* with BSS, an emerging pathogenesis in reef fishes.

The existence of multiple, genetically distinct species within the geographic distribution encompassed by descriptions of *S. expansus* casts morphometric variation in descriptions of this species in a new light. For example, measurements provided by Jägerskiöld (1904, in Egypt) are mostly smaller than those of Creplin (1842, likely in Germany). In France, Dubois (1960) reported worms smaller than in both these prior accounts, yet oral suckers and internal organs were larger. Next, Foronda *et al*. (2009) reported even shorter body lengths in worms from the Canary Islands than Dubois (1960), along with eggs highly variable in size. Recently, González-García *et al*. (2023) reported worms from Mexico smaller than those described by Creplin (1842), Jägerskiöld (1904) and Dubois (1960). All of these authors attributed such differences to morphological variability within *S. expansus*, but the inclusion of multiple species in these descriptions of geographically widespread specimens seems likely to have contributed to this morphometric variation.

Even without the species diversity revealed by recent molecular studies, the cosmopolitan range of *S. expansus* implied by records in the literature (reviewed by Kohl *et al*., 2019) is questionable considering host distributions. While osprey definitive hosts conduct impressive latitudinal migrations, the American and European osprey constitute genetically distinct subspecies with flyways that do not overlap (Ferguson-Lees and Christie, 2001; Monti *et al*., 2015; Monti, 2021). Thus, transatlantic distribution of worms by osprey is unlikely. Mackrill (2023) emphasizes the unprecedentedness of a recent transatlantic journey from Scotland to Barbados by 1 osprey, speculating that the bird rested on oceangoing boats. Transatlantic transport of *Scaphanocephalus* in other migratory bird species is also unlikely, due to the high specificity for osprey. Another way that *S. expansus* could theoretically maintain population connectivity across the Atlantic is *via* transport of metacercariae in migrating fish hosts, but this also seems improbable. Metacercariae of *Scaphanocephalus* are recorded mainly in reef-associated fishes poorly adapted for long-distance migration across pelagic habitats. Transatlantic distributions are reported in just 116/2605 (4%) of reef fish species (Floeter *et al*., 2008; see also Evans *et al*., 2020). Recent molecular studies (González-García *et al*., 2023; Cohen-Sánchez *et al*., 2023*b*) encountered metacercariae of *Scaphanocephalus* in fish species (*X. novacula*, *M. curema*) reported on both sides of the Atlantic in resources such as FishBase (Froese and Pauly, 2023). However, both these fishes consist of geographically disjunct, cryptic species (Durand and Borsa, 2015; Nirchio *et al*., 2019). Overall, given the distribution and mobility of known hosts, the parasites described from osprey in Germany by Creplin (1842) are unlikely to be conspecific with specimens in North America and the western Atlantic; indeed, any individual species of *Scaphanocephalus* distributed on both sides of the Atlantic seems doubtful. This view is in keeping with the general tendency of molecular data to reveal multiple, geographically isolated species of digeneans within samples of allegedly cosmopolitan species (Locke *et al*., 2021).

These 2 considerations – unclear morphological foundations in *S. expansus*, and disjunct host assemblages across the Atlantic – influence our interpretation of phylogenetic results in *Scaphanocephalus*. Essentially, while 28S data indicate 3 species, we argue that 5 species are more likely present among currently sequenced specimens, for 2 reasons. First, 28S may be identical in species of digeneans distinguishable through life history, morphology and other markers, and, secondly, 2 of the 28S lineages in *Scaphanocephalus* have implausible transoceanic distributions. Examples of digenean species that are poorly distinguishable with 28S and other commonly used nuclear ribosomal markers include members of *Hysteromorpha* (Locke *et al*., 2018), *Pseudoheterolebes* (Martin *et al*., 2018), *Bivesicula* (Cribb *et al*., 2022), *Posthodiplostomum* (Achatz *et al*., 2021) and *Transversotrema* (Cutmore *et al*., 2023). In neither *S. robustus* n. sp. nor *Scaphanocephalus* sp. ne2 do the 28S data provide sufficiently strong support for conspecificity of specimens separated by oceanic barriers. In the case of *S. robustus* n. sp., American isolates can be distinguished from Palearctic species of *Scaphanocephalus* both morphologically and by 5–7% divergence in CO1, which is higher than usually observed within species of digeneans (e.g. Vilas *et al*., 2005).

Among the 3 lineages of 28S rDNA recovered in 33 specimens, *Scaphanocephalus* sp. ne1 has been recovered in the Gulf of Mexico, Caribbean and North America, but not the Old World, where Creplin (1842) described *S. expansus*. González-García *et al*. (2023) identified adult specimens of this species as *S. expansus*, based on morphological similarity to prior descriptions. However, we doubt these specimens correspond to *S. expansus* because, as mentioned above, trans-Oceanic distributions are unlikely in species of *Scaphanocephalus*; no molecular evidence supports such a distribution in *Scaphanocephalus* sp. ne1; and the likely inclusion of multiple species in the morphological concept of *S. expansus* s.l. weakens identification on morphological grounds.

The mitochondrial genome phylogeny provides strong and independent support of both early and more recent systematic hypotheses. In creating *Scaphanocephalus*, Jägerskiöld (1904) noted its close relationship to *Cryptocotyle*. Kohl *et al*. (2019) were the first to support this close relationship using molecular data, namely partial 28S. At the family level, the paraphyly newly observed here in the evolution of mitochondrial genomes, with the Opisthorchidae nested within Heterophyidae, was first reported by Olson *et al*. (2003) and has emerged repeatedly in increasingly taxon-dense analyses of rDNA subunits by Thaenkham *et al*. (2011), Kuzmina *et al*. (2018), Pérez-Ponce de León and Hernández-Mena (2019), Tatonova and Besprozvannykh (2019) and Sokolov *et al*. (2022).

The new molecular data provided herein will be useful for resolving the status of both the unidentified lineages already encountered, and of *S. adamsi*, considered a synonym of *S. expansus* by Yamaguti (1942). The rDNA operon, mitochondrial genome and CO1 sequences will facilitate surveys and phylogenetic work based on commonly used markers heretofore unused in *Scaphanocephalus* (e.g. 18S, ITS, NAD1). For example, these data can help identify the first intermediate host of *Scaphanocephalus.* The use of littorinoid and truncatelloid snails by members of *Cryptocotyle* (Stunkard, 1930; Rothschild, 1938; Wootton, 1957; Tatonova and Besprozvannykh, 2019) suggests coastal-dwelling members of these clades may serve as hosts for *Scaphanocephalus*. Thus far, all known life cycles of opisthorchioid trematodes, which include the heterophyids, opisthorchids and cryptogonimids, involve snails in 1 of 3 superfamilies: Cerithioidea, Truncatelloidea, and Littorinoidea (with *Cryptocotyle* as the sole representative using this last superfamily) (Cribb *et al*., 2001).

Identification of a gastropod intermediate host of *Scaphanocephalus* may shed light on regional variation in BSS epidemiology, which is a research priority due to potential effects on algal grazers that contribute to coral reef health (Mumby *et al*., 2007; Burkepile and Hay, 2008; Kohl *et al*., 2019). BSS appears to have increased in prevalence in the Caribbean in recent decades (Elmer *et al*., 2019), and Cohen-Sánchez *et al*. (2023*a*, 2023*b*) also note a recent increase in reports in the Mediterranean. In most of the world, the cause of BSS has been treated as a single, cosmopolitan species, *S. expansus*, but we argue that the global distribution of definitive (Monti *et al*., 2015) and intermediate hosts (Costello *et al*., 2017) suggests *S. expansus*, described initially from Germany, is probably limited to Eurasia and Africa. This predicted continental separation of species in *Scaphanocephalus* is supported by newly obtained CO1 distinguishing a new American species, *S. robustus*, from a European species with matching 28S. To clarify the status of other species of *Scaphanocephalus*, mitochondrial sequences are needed from morphologically characterized adults of *Scaphanocephalus* in Europe. Such data would resolve the conflict between our conclusions and those of Al-Salem *et al*. (2021), who maintained that identical 28S in the Persian Gulf and Caribbean indicated a cosmopolitan species; and of González-García *et al*. (2023), who concluded the distribution of *Scaphanocephalus* ‘*expansus*’ to be cosmopolitan, based on adult morphology.

## Supporting information

Locke et al. supplementary material Locke et al. supplementary material

Locke et al. supplementary material 2 Locke et al. supplementary material

## Data Availability

Sequence data are available at GenBank accessions: PP436423-40 (partial 28S); PP456658–84 (partial CO1); PP577105 (mitochondrial genome); PP430581 (rDNA operon). Specimen vouchers are deposited in the Museum of Southwestern Biology (MSB:Para:49134–46).

## Supplementary material

The supplementary material for this article can be found at https://doi.org/10.1017/S0031182024000647.
