# Supplementary material for: Expanding on expansus: a new species of Scaphanocephalus from North America and the Caribbean based on molecular and morphological data
Source: Parasitology. 2024 May 21;151(7):679–91. doi: 10.1017/S0031182024000647 (PMC11474018; doi:10.1017/S0031182024000647)
Supplement: Locke et al. supplementary material [file S0031182024000647sup001.pdf]

Supplementary Figure 1
Phylogenetic analysis of 28 partial 28S sequences of *Scaphanocephalus* in the present (16 sequences, PP436423-40, PP430581) and prior studies (12 sequences). The Maximum Likelihood topology is shown and nodes are annotated with posterior probability (Bayesian Inference) / bootstrap support (1000 replicates in Maximum Likelihood). The trimmed alignment was 1048 nt in length.

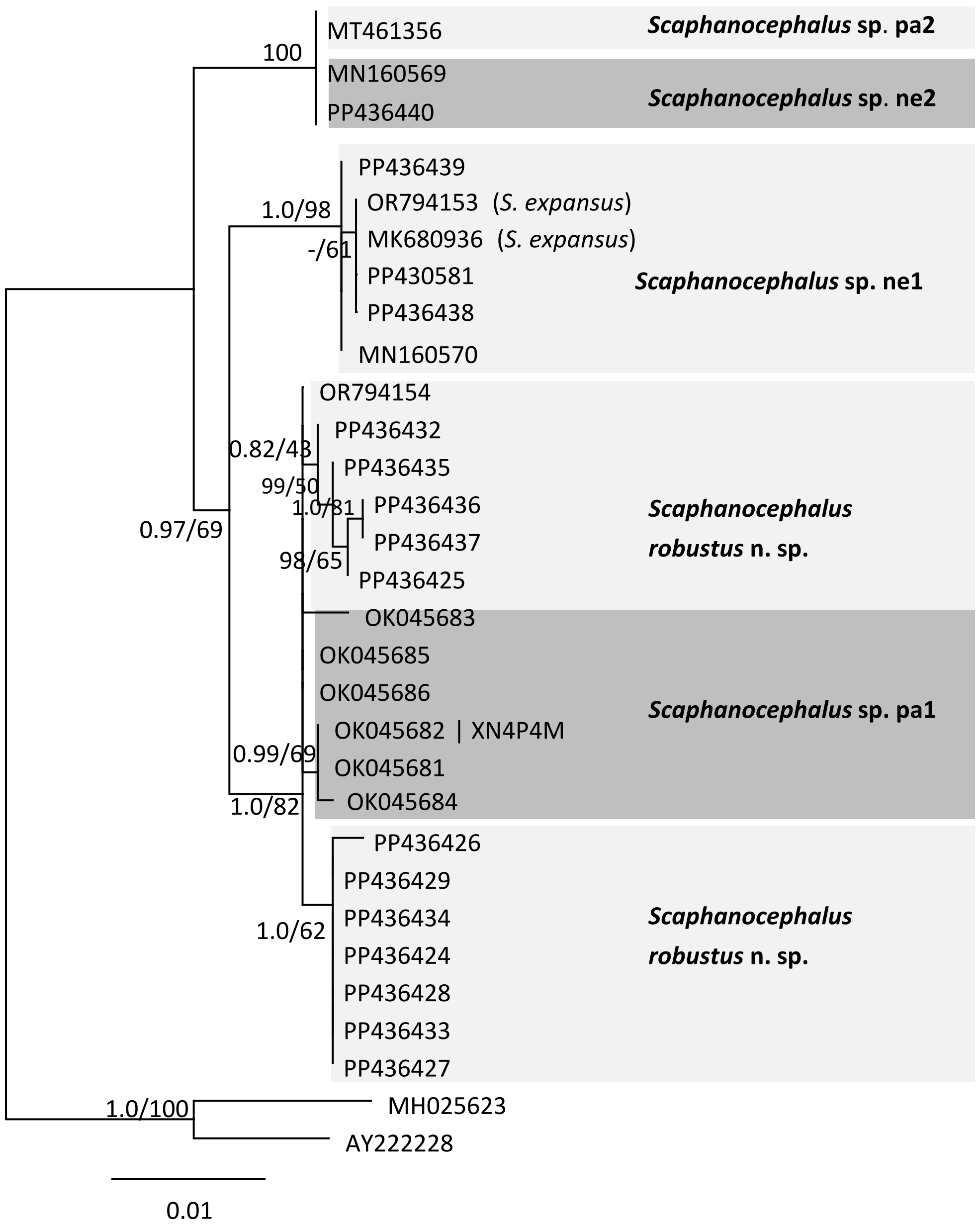

Supplementary Figure 2

Phylogenetic analysis of a 415-nt alignment of 26 partial CO1 sequences of *Scaphanocephalus* generated in the present study. The Bayesian inference topology is shown with nodes annotated with posterior probability (Bayesian Inference, BI) / bootstrap support (1000 replicates in ML). The ML tree (not shown) was generated using substitution model GTR+I; the BI tree using HKY+I.

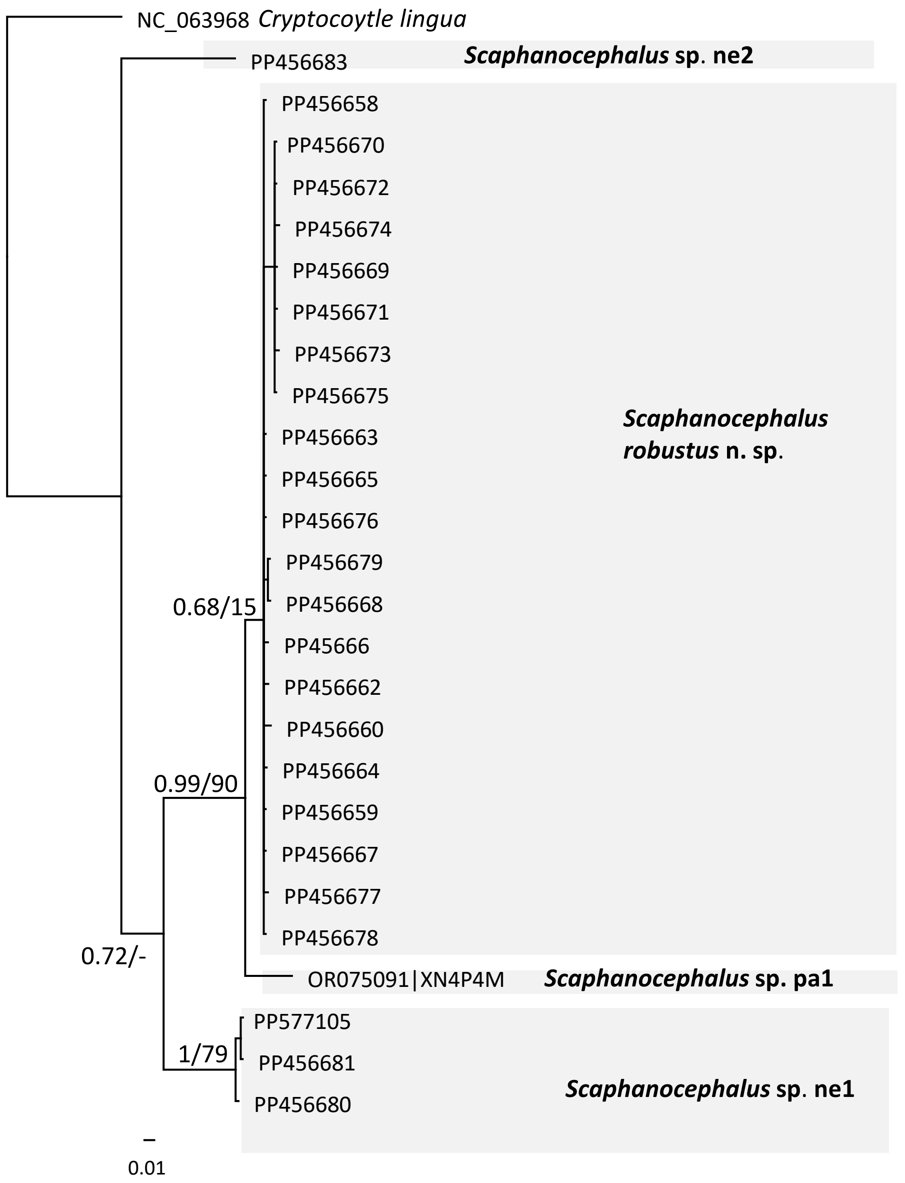

Supplementary Figure 3A
Schematic of aligned mitochondrial data generated in this study. Region in grey shaded box is expanded in **Supplementary Figure 3B**. Sequencing methods: I=Illumina, M=Minion, S=Sanger. Shapes show coding genes (yellow), tRNA (pink), and rRNA (red).

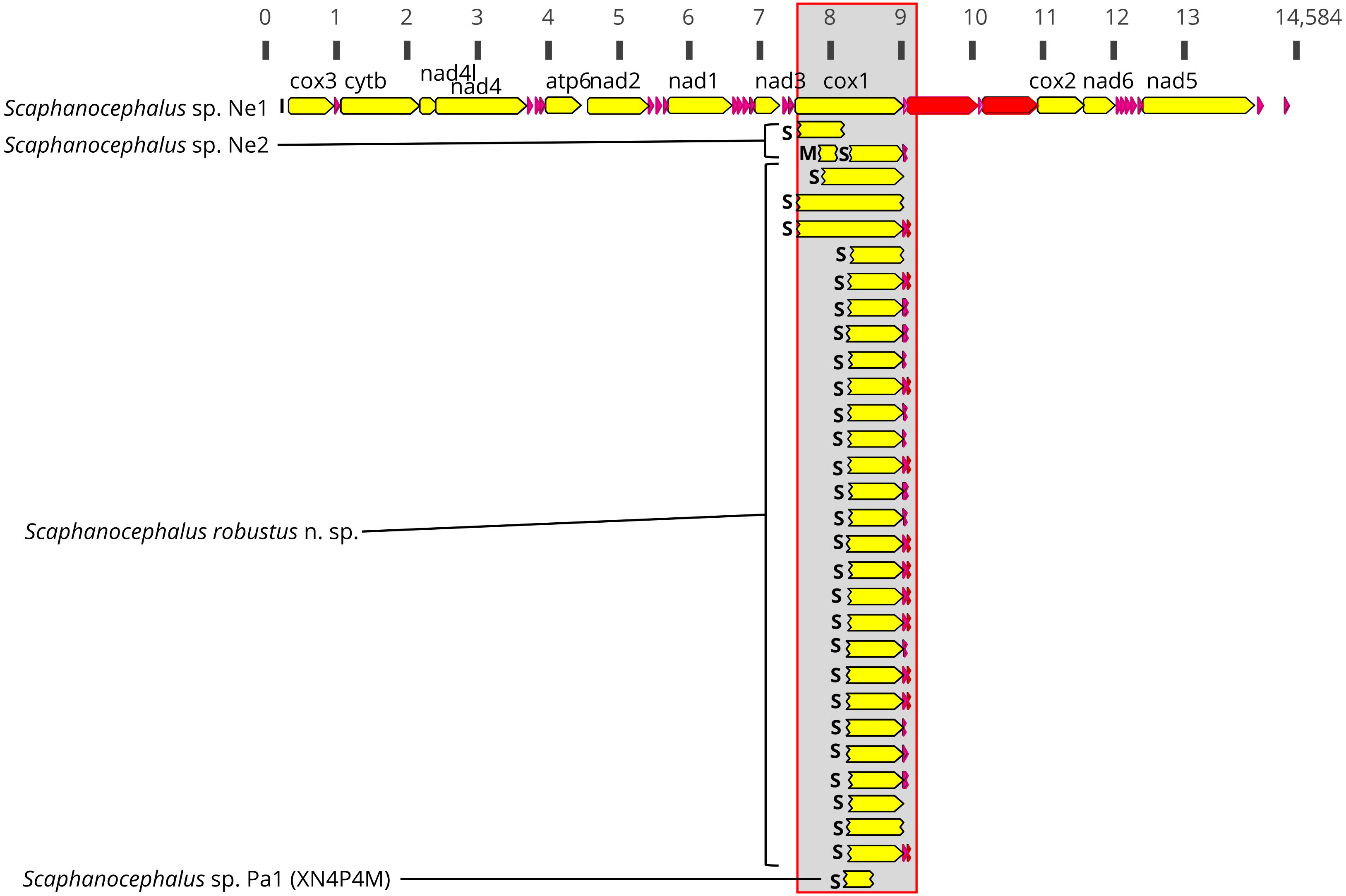

Supplementary Figure 3B

Detailed schematic of aligned cytochrome *c* oxidase I sequences generated in this study. Grey bars represent identity with a consensus sequence (not shown) and vertical marks in grey bars indicate differences from the consensus. Details in red box shown in Supplementary Figure S2C. Sequencing methods: I=Illumina, M=Minion, S=Sanger. Shapes show coding genes (yellow), tRNA (pink), and rRNA (red).

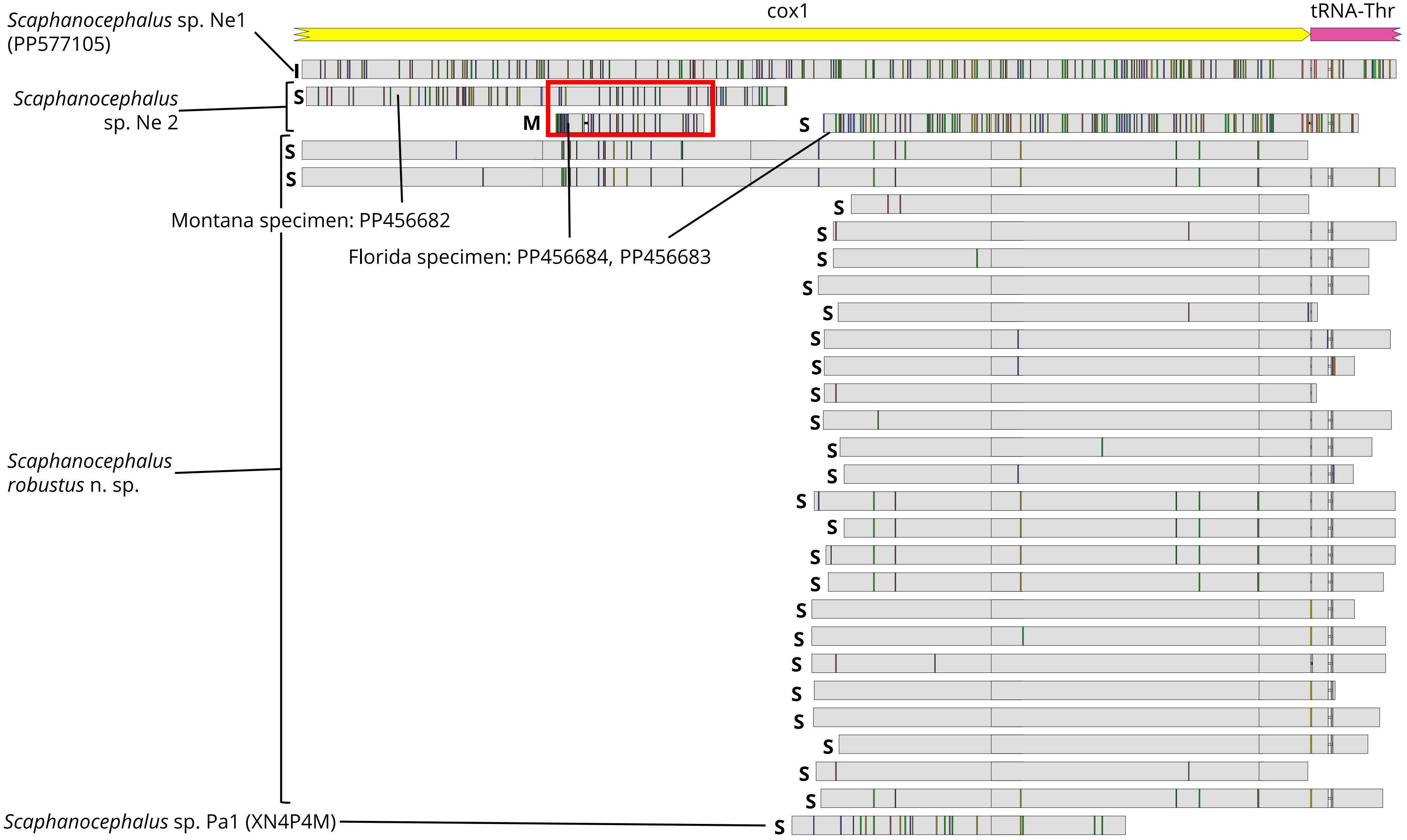

Supplementary Figure 3C-D

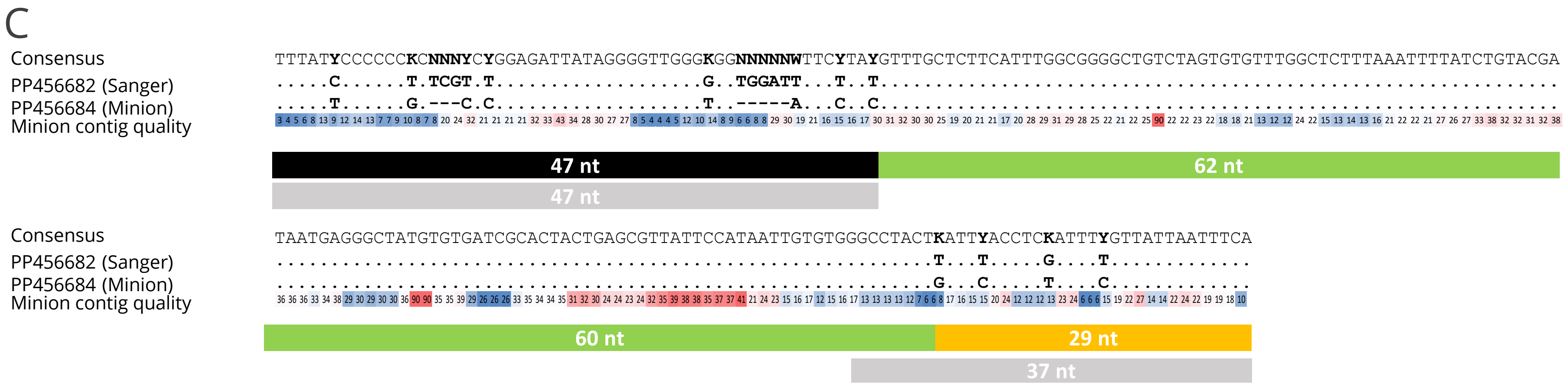

Supplementary Figure 3C. Aligned Minion and Sanger sequences of cytochrome *c* oxidase 1 from two specimens of *Scaphanocephalus* sp. Ne2 Quality of the Minion contig is below the sequence and color coded from dark blue (low) to bright red (high). Colored bars below alignment highlight regions with different quality scores summarized in Fig. S2D. Grey bars indicate regions automatically trimmed by default parameters with Geneious. Detail from Supplementary Figures 3A and B.

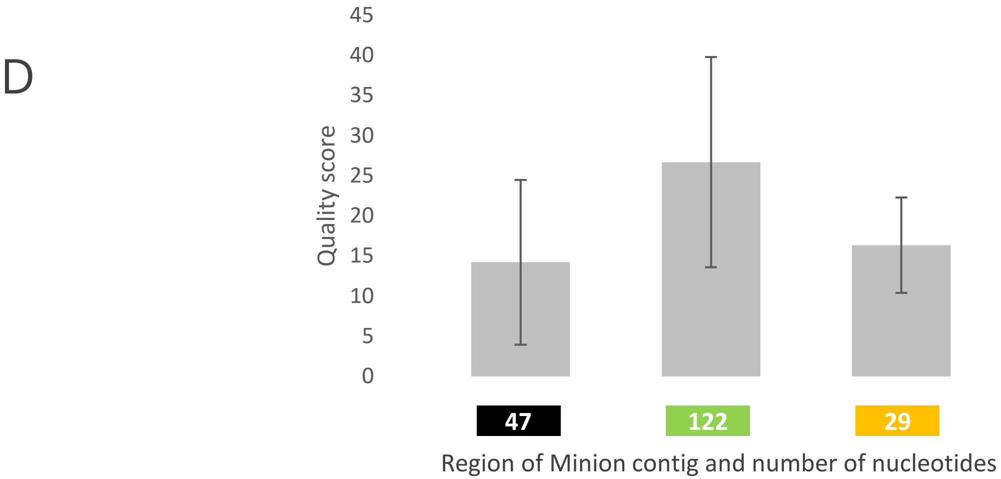

Supplementary Figure 3D. Mean (±SD) quality scores of three regions of the Minion contig of cytochrome *c* oxidase 1 from a specimen of *Scaphanocephalus* sp. Ne2. The regions are color-coded and labelled with number of nucleotides as in Fig 3C.
